# Can Magnetic Resonance Radiomics Analysis Discriminate Parotid Gland Tumors? A Pilot Study

**DOI:** 10.3390/diagnostics10110900

**Published:** 2020-11-03

**Authors:** Michela Gabelloni, Lorenzo Faggioni, Simona Attanasio, Vanina Vani, Antonio Goddi, Sara Colantonio, Danila Germanese, Claudia Caudai, Luca Bruschini, Mariella Scarano, Veronica Seccia, Emanuele Neri

**Affiliations:** 1 Master in Oncologic Imaging, Diagnostic and Interventional Radiology, Department of Translational Research, University of Pisa, Via Roma, 67, 56126 Pisa, Italy; mgabelloni@sirm.org (M.G.); emanuele.neri@med.unipi.it (E.N.); 2 Diagnostic and Interventional Radiology, Department of Translational Research, University of Pisa, Via Roma, 67, 56126 Pisa, Italy; simona.attanasio@med.unipi.it (S.A.); vanina.vani87@gmail.com (V.V.); goddi.anto@gmail.com (A.G.); 3 Institute of Information Science and Technologies “A. Faedo” of the National Research Council of Italy (ISTI-CNR), 56124 Pisa, Italy; sara.colantonio@isti.cnr.it (S.C.); danila.germanese@isti.cnr.it (D.G.); claudia.caudai@isti.cnr.it (C.C.); 4 Otolaryngology, Audiology, and Phoniatric Operative Unit, Department of Surgical, Medical, Molecular Pathology, and Critical Care Medicine, Azienda Ospedaliero Universitaria Pisana, University of Pisa, 56124 Pisa, Italy; l.bruschini@gmail.com (L.B.); mariella.scarano88@gmail.com (M.S.); veronicaseccia@gmail.com (V.S.)

**Keywords:** head and neck cancer, parotid neoplasm, artificial intelligence, Warthin tumor, adenoma, pleomorphic

## Abstract

Our purpose is to evaluate the performance of magnetic resonance (MR) radiomics analysis for differentiating between malignant and benign parotid neoplasms and, among the latter, between pleomorphic adenomas and Warthin tumors. We retrospectively evaluated 75 T2-weighted images of parotid gland lesions, of which 61 were benign tumors (32 pleomorphic adenomas, 23 Warthin tumors and 6 oncocytomas) and 14 were malignant tumors. A receiver operating characteristics (ROC) curve analysis was performed to find the threshold values for the most discriminative features and determine their sensitivity, specificity and area under the ROC curve (AUROC). The most discriminative features were used to train a support vector machine classifier. The best classification performance was obtained by comparing a pleomorphic adenoma with a Warthin tumor (yielding sensitivity, specificity and a diagnostic accuracy as high as 0.8695, 0.9062 and 0.8909, respectively) and a pleomorphic adenoma with malignant tumors (sensitivity, specificity and a diagnostic accuracy of 0.6666, 0.8709 and 0.8043, respectively). Radiomics analysis of parotid tumors on conventional T2-weighted MR images allows the discrimination of pleomorphic adenomas from Warthin tumors and malignant tumors with a high sensitivity, specificity and diagnostic accuracy.

## 1. Introduction

Salivary gland tumors are relatively rare with an annual worldwide incidence ranging from 0.05 to 2 per 100,000 individuals [1]. Almost 80% of tumors affect parotid glands and most of them are benign (80%). Of the latter, the most frequent neoplasm is the pleomorphic adenoma followed by the Warthin tumor [2]. Among malignant tumors, the overall most frequent one is the mucoepidermoid carcinoma whereas the adenoid cystic carcinoma is the commonest cancer in the submandibular and minor salivary glands.

Salivary gland tumors’ early symptoms such as palpable lesions and pain are non-specific and can be present in both benign and malignant lesions; the presence of facial paralysis, which is a sign of malignancy, appears later in the clinical course of the patient and cannot be used as an early sign of alert of malignancy for the appropriate management of the lesion.

Therefore, pre-operative imaging is a stronghold of major importance for the correct framing of a salivary gland lesion in a surgical or non-surgical setting and for the level of clinical priority. While conventional imaging features can provide clues to the diagnosis, the radiological appearances of parotid masses may considerably overlap and open biopsy is not recommended in light of the risk of local complications (pain, bleeding, facial nerve damage) and more importantly of the tumor seeding in the operative field. Despite being minimally invasive, fine needle aspiration cytology (FNAC) shares the aforementioned limitations and can be hindered by anatomic factors such as tumor location in the deep parotid lobe [3]. Moreover, FNAC shows a highly variable level of accuracy and ongoing efforts have been made to find new elements (e.g., in the field of proteomics) that could lead to a more accurate pre-surgical diagnosis [4].

Owing to its good spatial resolution and excellent soft tissue contrast resolution, magnetic resonance imaging (MRI) is the imaging modality of choice for local staging, providing accurate and comprehensive information about disease location and extent (including, for example, the presence of perineural spread and bone invasion) and some characteristic MRI features have been described that could serve as clues to the diagnosis. However, conventional MRI has an overall limited performance in differentiating benign from malignant salivary gland tumors and between the various types of benign tumors [5]. Consequently, there is a growing interest in the development of advanced approaches that can extract quantitative data from standard MR images to improve diagnostic accuracy and possibly allow individualized patient treatment and outcome prediction [6,7]. In this context, radiomics is one of the most innovative fields of oncologic imaging [8] and radiomics texture features have been evaluated for various neoplasms including, for example, breast cancer [9], non-small cell lung cancer [10], lung adenocarcinoma [11] and prostate adenocarcinoma [12].

Our purpose is to evaluate the performance of MR radiomics analysis for differentiating between malignant and benign parotid neoplasms and, among the latter, between pleomorphic adenomas and Warthin tumors.

## 2. Materials and Methods

This was a retrospective study involving 75 patients (45 male, 30 female, age 16–81, median 58.4) with parotid gland lesions who underwent a head and neck MRI examination at our referral center for disease staging and surgical planning between March 2010 and July 2018. Written informed consent to MR imaging for the diagnostic workup of parotid lesions was obtained from all patients and institutional review board approval was waived due to the retrospective nature of the study. Seventy-five parotid masses were detected, of which 14 turned out to be malignant tumors on histopathological analysis (three mucoepidermoid carcinomas, two squamous cell carcinomas, two adenoid cystic carcinomas, one myoepithelioma, one acinar cell carcinoma, one lymphoma and four metastases) and 61 were benign tumors (32 pleomorphic adenomas, 23 Warthin tumors and six oncocytomas).

All MRI examinations were carried out on a commercial 1.5-Tesla whole body scanner (Signa HDxt, General Electric, Milwaukee, WI, USA) with a dedicated 16-channel neurovascular coil.

**Figure 1:**
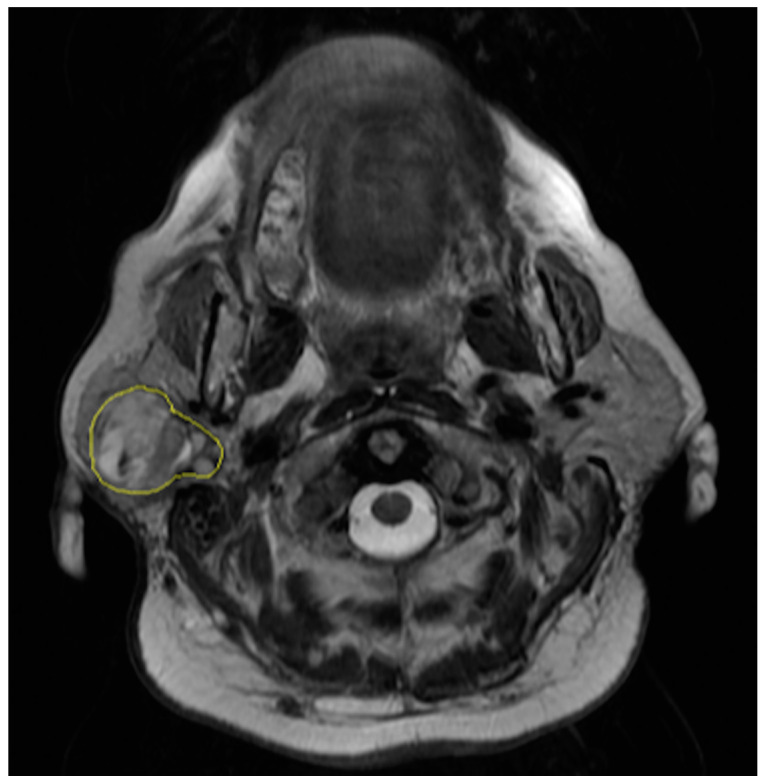
Segmentation of axial T2-weighted magnetic resonance (MR) image of a 61-year-old woman with a pleomorphic adenoma of the right parotid gland.

The regions of interest were manually drawn by a radiologist with eight years of experience in oncologic and head and neck imaging who was blinded to the results of the pathological examinations. The radiologist contoured the outer edge of the entire tumor slice by slice on axial fast spin-echo T2-weighted images (Figure 1). Contouring was performed so as to cover the maximum extent of the tumor without exceeding the lesion border.

MRI scan parameters for axial fast spin-echo T2-weighted images were the following: TR/TE 4580/108 ms, slice thickness 4.5 mm, matrix 352 × 288, FOV 25 cm. Following segmentation, texture analysis was performed by using the QUIBIM Precision platform (QUIBIM SL, Valencia, Spain). For each lesion, a total of 29 quantitative radiomics features were automatically generated including a gray level histogram and co-occurrence matrix analysis according to the software settings.

Data were normalized to obtain values ranging between 0 and 1. Prior to normalization, outliers were checked and those that were not significant were removed. Oncocytomas were excluded from the analysis due to their small number.

Quantitative data were displayed visually using boxplots. The unpaired two-sample Wilcoxon rank-sum test was used to seek MR texture features that allowed the differentiation of malignant from benign parotid tumors and, among the latter, pleomorphic adenomas from Warthin tumors. The Pearson correlation coefficient was calculated to explore linear correlations between features and eliminate redundant ones in the following way: when a pair of features showed a Pearson coefficient higher than 90%, only the feature with the lower Wilcoxon rank test *p*-value was retained.

A receiver operating characteristics (ROC) curve analysis was performed to find the threshold values for the most discriminative features and determine their sensitivity, specificity and area under the ROC curve (AUROC). The most discriminative features were used to train a support vector machine classifier and the ability of the latter to correctly discriminate between the various disease conditions was assessed in terms of sensitivity, specificity and diagnostic accuracy.

## 3. Results

### 3.1. Malignant Versus Benign Parotid Tumors

The features autocorrelation value, cluster shade value, sum average value, skewness value, gray level mean, gray level standard deviation, gray level median, gray level p25 and gray level p75 were significantly different between malignant and benign parotid tumors. Of them, non-redundant features were autocorrelation value, cluster shade value, skewness value, gray level mean and gray level standard deviation (Table 1).

**Figure 2:**
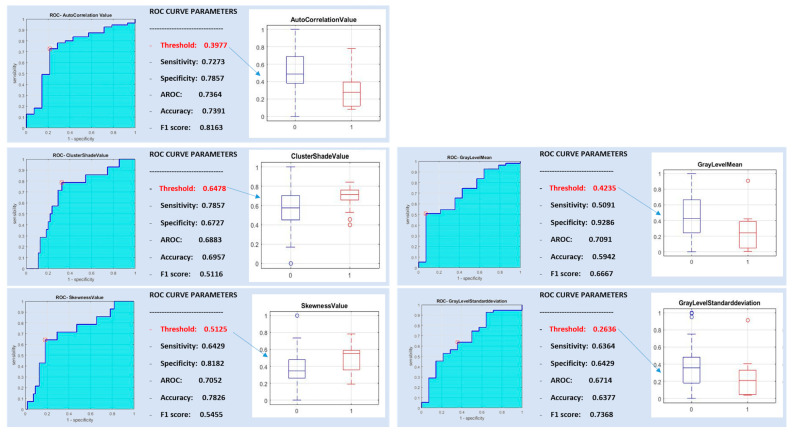
Receiver operating characteristics (ROC) curve analysis for benign versus malignant parotid tumors.

In Figure 2, the ROC curve analysis is represented for the post-correlation of the five significant features discriminating between benign and malignant tumors.

A support vector machine classifier was trained with the five non-redundant most discriminative features and with all subsets of them. The best classification performance was obtained with a radiomic signature consisting of a combination of autocorrelation value, skewness value and gray level mean, yielding high specificity (0.8857) but low sensitivity and diagnostic accuracy (0.2941 and 0.5942, respectively).

### 3.2. Pleomorphic Adenoma Versus Warthin Tumor

The features autocorrelation value, cluster shade value, maximum probability value, sum average value and skewness value were significantly different between pleomorphic adenomas and Warthin tumors. The autocorrelation value and sum average value showed a correlation higher than 90% (Table 2).

**Figure 3:**
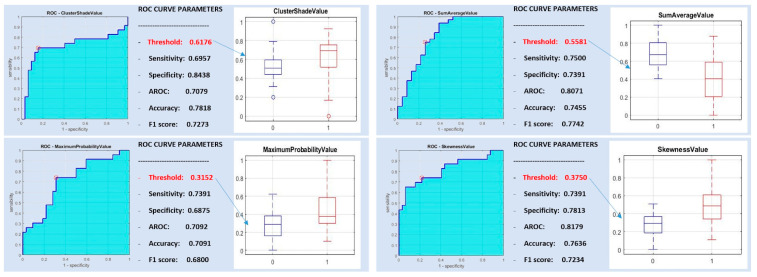
ROC curve analysis for pleomorphic adenoma versus Warthin tumors.

Figure 3 shows good AUROC, sensitivity and specificity results for the four post-correlation significant features differentiating pleomorphic adenoma from Warthin tumors: cluster shade value, maximum probability value, sum average value and skewness value.

A support vector machine classifier was trained with all of the aforementioned most discriminative features and with all subsets of them, yielding sensitivity, specificity and diagnostic accuracy as high as 0.8695, 0.9062 and 0.8909, respectively.

### 3.3. Pleomorphic Adenoma Versus Malignant Parotid Tumors

Several features (i.e., autocorrelation value, cluster shade value, sum average value, skewness value, gray level mean, energy value, maximum probability value, gray level p25 and gray level p75) were significantly different between pleomorphic adenomas and malignant parotid tumors. Correlations greater than 90% were found between the sum average value and the autocorrelation value, between the maximum probability value and the energy value, between the gray level p25 and the gray level mean, between the gray level p75 and the gray level mean and between the gray level p25 and the gray level p75 (Table 3).

**Figure 4:**
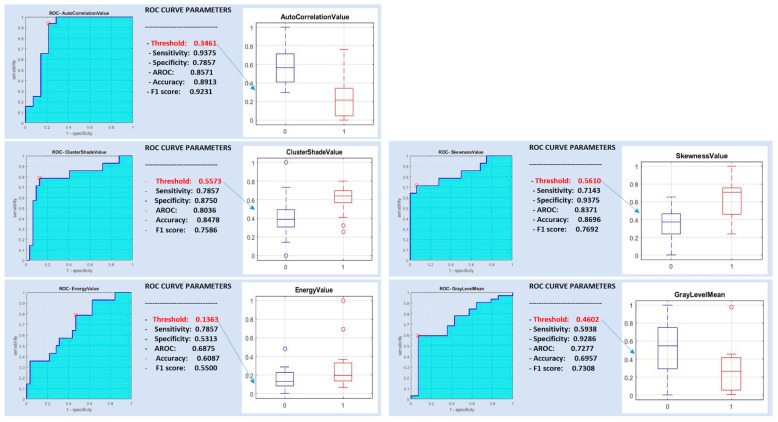
ROC curve analysis for pleomorphic adenoma versus malignant tumors.

A support vector machine classifier was trained with the above-mentioned five non-redundant features (Figure 4) and with all subsets of them.

The best classification performance differentiating pleomorphic adenoma from malignant tumors was obtained with a radiomic signature consisting of a combination of the autocorrelation value and skewness value, yielding a sensitivity, specificity and diagnostic accuracy of 0.6666, 0.8709 and 0.8043, respectively.

### 3.4. Warthin Tumor Versus Malignant Parotid Tumors

Only the features cluster prominence value and sum of square variance value were significantly different between Warthin tumors and malignant parotid tumors and showed a >90% correlation with each other (Table 4).

**Figure 5:**
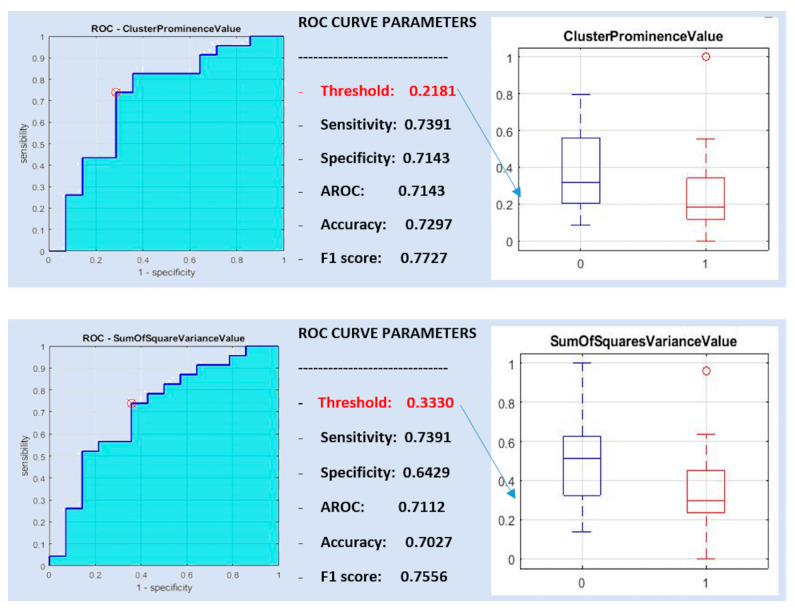
ROC curve analysis for Warthin versus malignant tumors.

Both features (cluster prominence value and sum of square variance value) had a sensitivity of 0.7391, whereas they showed a specificity of 0.7143 and 0.6429 and a diagnostic accuracy of 0.7143 and 0.7112, respectively (Figure 5).

A support vector machine classifier was trained with the single features and with both. The best classification performance in differentiating between Warthin and malignant tumors was obtained with the cluster prominence value, yielding poor vales of sensitivity, specificity and diagnostic accuracy of 0, 0.6216 and 0.6216, respectively.

## 4. Discussion

The development of radiomics has opened new scenarios owing to the possibility of non-invasively assessing features (such as those derived from the analysis of tissue texture) that are not visible by the human eye, potentially allowing for a more accurate characterization of tumors in their entirety as well as for predicting patient outcome and for individualized treatment planning [13]. As a matter of fact, the number of published articles on the topic has been growing steadily over the last years and several studies have shown associations between radiomics features and tissue structure that can be useful for the diagnosis and management of various tumors [14].

In our study we found that radiomics features based on a histogram and gray level co-occurrence matrix (GLCM) can help discriminate between different conditions such as a pleomorphic adenoma versus a Warthin tumor, a pleomorphic adenoma versus malignant parotid tumors and benign versus malignant parotid tumors. The histogram of an image represents how many pixels with the same given gray level value are present in an image. Kurtosis and skewness are histogram-based features, the latter being an indicator of the asymmetry of the distribution of gray levels with respect to the average value within the region of interest. Higher skewness values have been associated with angiogenesis in several neoplasms, which in turn is a hallmark of tumor growth and metastasis [10,15,16]. This could explain our finding of higher skewness values in malignant than in benign parotid tumors.

Several authors reported that Warthin tumors have a higher grade of vascularity than a pleomorphic adenoma [17,18] and exhibit a marked central perfusion while pleomorphic adenomas tend to exhibit a higher marginal perfusion [14]. This could explain our finding of higher skewness values in Warthin tumors than in pleomorphic adenomas.

Other statistically significant features that allow differentiation between the various parotid lesions were GLCM-based. The GLCM (also called a second-order histogram) can be used to quantify the texture of an image by calculating how often pixel pairs with specific values and with a given spatial relationship occur in an image and then extracting statistical measures from the matrix that is obtained [19]. The autocorrelation value depends on the degree of correlation between a variable in a given region and the same variable in a nearby region and, hence, is a descriptor of heterogeneity or clustering in an image [20,21]. In our study, the autocorrelation value was found to be able to discriminate between benign and malignant tumors and between pleomorphic adenomas on the one hand versus malignant or Warthin tumors on the other hand. This could be explained by higher tissue homogeneity in benign than in malignant tumors. In fact, malignant tumors are more commonly composed of cells with a different and heterogeneous size and texture, leading to a lower autocorrelation value as a result of different values of a given texture-related variable within neighboring regions whereas benign tumors would show higher autocorrelation values due to having a more regular and homogeneous structure. Among benign parotid tumors, Warthin tumors seem to have a higher heterogeneity than pleomorphic adenomas, possibly resulting in lower autocorrelation values [18].

The cluster shade value is a second-order feature that can be derived from the GLCM to obtain a measure of matrix asymmetry, with higher values corresponding to greater asymmetry. In our study, the cluster shade value was higher in malignant than in benign tumors, possibly in relation to the higher structural heterogeneity of the former. Likewise, higher tissue heterogeneity could explain our finding of higher cluster shade values in Warthin tumors compared with pleomorphic adenomas and in malignant tumors compared with pleomorphic adenomas [22].

We also found higher maximum probability values in malignant than in benign tumors as well as in Warthin tumors than in pleomorphic adenomas and in malignant tumors than in pleomorphic adenomas. The maximum probability value is an indicator of how many times a given combination of gray levels occurs more frequently within a region of interest and it has been found to correlate with worse survival in a study on breast cancer imaging features by Fan et al. [23].

While we were able to find radiomics features that allowed the distinguishing of pleomorphic adenomas from malignant tumors and Warthin tumors, we failed to differentiate the latter from one another using the same features. This could be due to the greater tissue heterogeneity and vascularity of Warthin tumors compared with pleomorphic adenomas, which make it more similar to malignant tumors and therefore more difficult to discriminate based on radiomics features that reflect such properties.

To our knowledge, this is the first study aimed at differentiating between various parotid tumors by means of T2-weighted MRI-based radiomics analysis. Fruehwald-Pallamar et al. assessed radiomics features of parotid neoplasms from pre- and post-contrast T1-weighted MR images in 38 patients (of whom seven had a malignant tumor and 31 had a benign mass, including 13 Warthin tumors and 11 pleomorphic adenomas) and found that, in general, the discrimination between benign and malignant tumors was more feasible than between pleomorphic adenomas and Warthin tumors [24].

Our study should be considered no more than exploratory due to a few limitations. First, our overall relatively small patient sample and the unequal distribution of the various tumor types could have prevented us from finding additional information and/or could have introduced a bias due to the higher prevalence of one or another tumor type. Second, radiomics analysis was carried out on image datasets without prior data normalization or pre-processing, possibly altering the radiomics features calculated from them. However, although such an error cannot be quantified precisely, we were still able to obtain data allowing for a useful differentiation between various types of parotid mass that are commonly encountered in clinical practice. Third, we used T2-weighted images for radiomics analysis so we cannot exclude a partial contribution to the skewness value (which reflects the average brightness of highlighted objects) from hyperintense cystic components. Moreover, the fact of using T2-weighted images only instead of contrast-enhanced T1-weighted images may have limited our ability to collect information directly related to neoangiogenesis. Finally, it was not possible to have an external validation using a test set, given the limited number of patients.

In conclusion, our findings show that radiomics analysis of parotid tumors on conventional T2-weighted MR images allows the discrimination of pleomorphic adenomas from Warthin tumors and malignant tumors with high sensitivity, specificity and diagnostic accuracy. Further investigation is warranted to test the validity of our findings on a broader patient sample and in more specific tumor types.

## Figures and Tables

**Table 1:** Values of MR radiomics features of benign versus malignant parotid tumors.

Radiomics Feature	*p*-Value
Autocorrelation Value	0.0068
Cluster Prominence Value	0.0698
Cluster Shade Value	0.0311
Contrast Value	0.2074
Correlation Value	0.6598
Difference Entropy Value	0.2860
Difference Variance Value	0.1817
Dissimilarity Value	0.2355
Energy Value	0.1675
Entropy Value	0.2794
Homogeneity Value	0.4247
Information Measure of Correlation 1 Value	0.1585
Information Measure of Correlation 2 Value	0.1262
Inverse Difference Value	0.4161
Maximum Probability Value	0.1867
Sum Average Value	0.0088
Sum Entropy Value	0.1225
Sum of Squares Variance Value	0.0515
Sum Variance Value	0.0632
Kurtosis Value	0.1023
Skewness Value	0.0188
Gray Level Mean	0.0167
Gray Level Standard Deviation	0.0497
Gray Level Median	0.0195
Gray Level p25	0.0203
Gray Level p75	0.0195
D2 D Value	0.3827
D3 D Value	0.1769
Volume Value	0.8055

Statistically significant features (*p* < 0.05) are highlighted in gray.

**Table 2:** Values of MR radiomics features of pleomorphic adenoma versus Warthin tumors.

Radiomics Feature	*p*-Value
Autocorrelation Value	0.0003
Cluster Prominence Value	0.1145
Cluster Shade Value	0.0093
Contrast Value	0.9117
Correlation Value	0.9932
Difference Entropy Value	0.7523
Difference Variance Value	0.9252
Dissimilarity Value	0.7265
Energy Value	0.0833
Entropy Value	0.1976
Homogeneity Value	0.4789
Information Measure of Correlation 1 Value	0.1643
Information Measure of Correlation 2 Value	0.1267
Inverse Difference Value	0.5003
Maximum Probability Value	0.0088
Sum Average Value	0.0001
Sum Entropy Value	0.7783
Sum of Squares Variance Value	0.1400
Sum Variance Value	0.1696
Kurtosis Value	0.8311
Skewness Value	<0.0001
Gray Level Mean	0.2565
Gray Level Standard Deviation	0.8578
Gray Level Median	0.2098
Gray Level p25	0.1145
Gray Level p75	0.2711
D2 D Value	0.4176
D3 D Value	0.3019
Volume Value	0.2494

Statistically significant features (*p* < 0.05) are highlighted in gray.

**Table 3:** Values of MR radiomics features of pleomorphic adenoma versus malignant tumors.

Radiomics Feature	*p*-Value
Autocorrelation Value	0.0001
Cluster Prominence Value	0.2101
Cluster Shade Value	0.0012
Contrast Value	0.2015
Correlation Value	0.5427
Difference Entropy Value	0.2881
Difference Variance Value	0.1932
Dissimilarity Value	0.2373
Energy Value	0.0462
Entropy Value	0.1295
Homogeneity Value	0.3457
Information Measure of Correlation 1 Value	0.3836
Information Measure of Correlation 2 Value	0.2881
Inverse Difference Value	0.3457
Maximum Probability Value	0.0346
Sum Average Value	0.0001
Sum Entropy Value	0.1295
Sum of Squares Variance Value	0.1357
Sum Variance Value	0.1420
Kurtosis Value	0.1020
Skewness Value	0.0003
Gray Level Mean	0.0154
Gray Level Standard Deviation	0.0878
Gray Level Median	0.144
Gray Level p25	0.0096
Gray Level p75	0.0154
D2 D Value	0.5912
D3 D Value	0.3836
Volume Value	0.6587

Statistically significant features (*p* < 0.05) are highlighted in gray.

**Table 4:** Values of MR radiomics features of Warthin tumors versus malignant tumors.

Radiomics Feature	*p*-Value
Autocorrelation Value	0.5008
Cluster Prominence Value	0.0319
Cluster Shade Value	0.7901
Contrast Value	0.3395
Correlation Value	0.9127
Difference Entropy Value	0.4066
Difference Variance Value	0.2800
Dissimilarity Value	0.3556
Energy Value	0.7901
Entropy Value	0.7901
Homogeneity Value	0.6725
Information Measure of Correlation 1 Value	0.0717
Information Measure of Correlation 2 Value	0.0717
Inverse Difference Value	0.6498
Maximum Probability Value	1
Sum Average Value	0.6498
Sum Entropy Value	0.2161
Sum of Squares Variance Value	0.0345
Sum Variance Value	0.0503
Kurtosis Value	0.2047
Skewness Value	0.8387
Gray Level Mean	0.0669
Gray Level Standard Deviation	0.0624
Gray Level Median	0.0938
Gray Level p25	0.1453
Gray Level p75	0.0878
D2 D Value	0.2662
D3 D Value	0.0938
Volume Value	0.9625

Statistically significant features (*p* < 0.05) are highlighted in gray.
